# Repeatability of High-Pressure Measurement in a Diesel Engine Test Bed

**DOI:** 10.3390/s20123478

**Published:** 2020-06-19

**Authors:** Tomasz Skrzek, Mirosław Rucki, Krzysztof Górski, Jonas Matijošius, Dalibor Barta, Jacek Caban, Janusz Zarajczyk

**Affiliations:** 1 Faculty of Mechanical Engineering, Kazimierz Pulaski University of Technology and Humanities in Radom, ul. B. Chrobrego 45, 26-600 Radom, Poland; t.skrzek@uthrad.pl (T.S.); krzysztof.gorski@uthrad.pl (K.G.); 2 Institute of Mechanical science, Vilnius Gediminas Technical University, J. Basanavičiaus str. 28, LT-03224 Vilnius, Lithuania; jonas.matijosius@vgtu.lt; 3 Faculty of Mechanical Engineering, University of Zilina, Univerzitna 1, 010 26 Zilina, Slovakia; dalibor.barta@fstroj.uniza.sk; 4 Faculty of Production Engineering, University of Life Sciences in Lublin, Głęboka 28, 20-612 Lublin, Poland; jacek.caban@up.lublin.pl

**Keywords:** high pressure, engine performance, engine test bed, measurement system, measurement uncertainty, repeatability

## Abstract

This paper addresses the issue of metrological accuracy of instantaneous in-cylinder pressure measurement in a diesel engine test bed. In studies, the central unit has been the single-cylinder AVL 5402 engine. The pressure measurement was performed with a sensor designed for thermodynamic analysis, and the results were related to the crank angle, where two rotations corresponding to the four-stroke working cycle were denoted as angles between −360° and +360°. The novelty of this paper is the proposition of how to perform a type A uncertainty estimation of the in-cylinder pressure measurement and to assess its repeatability. It was demonstrated that repeatability of the measurement during the ignition process was difficult to estimate because of the phenomena that cannot ensure the repeatability conditions. To solve the problem, two methods were proposed. In one method, the pressure was measured in the subsequent cycles immediately after the ignition was turned off, and in another method, the engine was driven by a starter. The latter method provided maximal pressure values much lower than during usual tests. The obtained repeatability of measured pressure was *%EV* = 0.4%, which proved high capability of the evaluated measurement system.

## 1. Introduction

The transport service sector is constantly focusing on the improvement of vehicle fuel consumption, which is the biggest part of the energy consumption in transport [1]. Despite the fact that vehicles with diesel engines have limitations when they enter city centers, the position of diesel engines seems to be still developing. Diesel engines are irreplaceable especially in the area of so-called off-road vehicles and for powering various types of machinery and equipment, as well as building machinery or agricultural tractors [2]. Compared with the spark-ignition engine, the compression-ignition engine has undisputable advantages, such as reliability, fuel efficiency, larger power range, longer lifetime and maintenance period, better torque characteristics, and higher power density [3].

Global reduction of petroleum resources and environmental issues related to the usage of internal combustion engines are leading to an increasing trend towards alternative energy sources [4,5,6,7]. To combat this problem, a large number of studies are being conducted worldwide [8,9,10,11,12], and in many cases, have been focused on in-cylinder pressure measurement. For example, Pan and co-authors developed a fast and reasonably accurate dynamic model that was based on a neural network nonlinear identification coupled with an unsupervised feature extraction methodology [13], however, they did not address the issue of measurement uncertainty. Similarly, in calculations of heat release based on the in-cylinder pressure derivative signal with application of the cut-off frequency of a low-pass filter, the uncertainty of the pressure measurement was not estimated [14]. In another work [15], the importance of accurate pressure measurement was highlighted, but the authors simplified the matter with the assumption from previous works that cylinder pressure measurements had an unknown but constant offset ∆*p*. Even a methodology proposed for estimating the exhaust temperature exclusively by relying on in-cylinder pressure signal, engine speed, and exhaust lambda, did not provide uncertainty estimation of pressure measurement [16]. In a study on an engine optimization model developed to fit the calculated in-cylinder pressure diagram to the experimental data [17], the latter were not given with uncertainty intervals. While the methodology was correct, the final accuracy of the model did not refer to the law of propagation of uncertainty, which indicated the need to propose a proper methodology of uncertainty estimation.

The assessment of uncertainty of measurement system for testing alternative fuels is a very complicated issue. Investigations of the cycle-to-cycle pressure variations for six rotational speeds of a crankshaft revealed that depending on the speed of rotation, the pressure variations can have a strongly periodic component or be intermittent [18]. Unlike many other measurement systems where reference devices or calibration standards can be applied [19,20], engine test rigs rely only on the accuracy of their components [21]. There has been no report, however, that addressed the repeatability of the pressure measurement system, and therefore our study aimed to fill this gap. The present study was designed to assess uncertainty and repeatability of the diesel engine performance test bed after replacement of the pressure sensor. On the basis of the experiences of other researchers who applied statistical methods to evaluate the whole system of vehicle speed measurement [22], a statistical analysis was assumed to be appropriate.

## 2. Materials and Methods

In the studies, the central unit has been the single-cylinder AVL 5402 engine. It has often been used for studies on combustion processes with biofuels [23], or various additions to diesel oil such as rape methyl esters [24] or ethyl-tert-butyl ether [25]. Other studies have used it for emission analysis, for example, for neat mahua oil biodiesel blended with different proportions of octanol [26] or focused on the chemical features of diesel exhaust particles [27]. Technical characteristics of the engine installed in the examined test bed are shown in Table 1.

**Figure 1:**
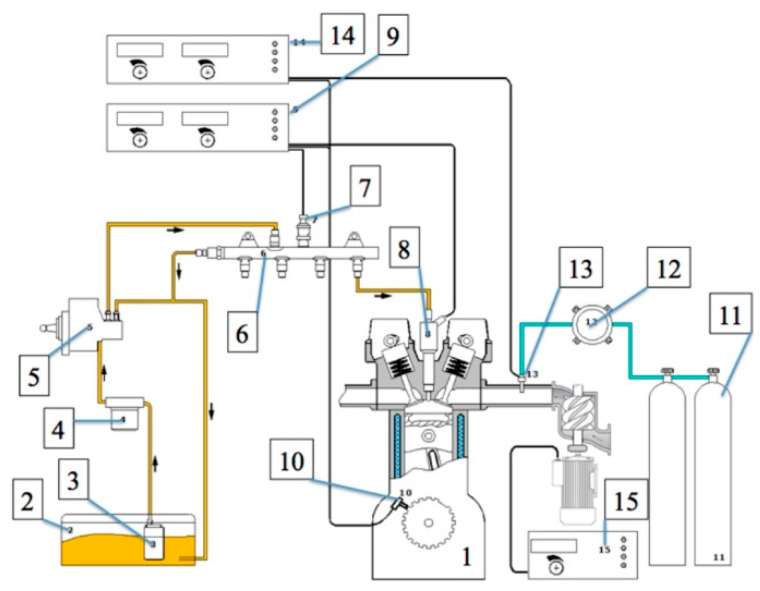
General scheme of the test bed supply (explanations in the text).

The measuring apparatus used for the tests was in conformity with the requirements of the following normative documents: Directive 1999/96/EC of the European Parliament and of the Council of 13 December 1999, Regulation (EC) no. 715/2007 of the European Parliament and of the Council of 20 June 2007, and Commission Regulation (EC) no. 692/2008 of 18 July 2008. The test bed consisted of the elements shown in Figure 1 as follows: (1) engine, (2) fuel tank, (3) electric fuel pump, (4) fuel filter, (5) high-pressure fuel pump, (6) rail, (7) fuel pressure sensor, (8) injector, (9) controller of common rail supply system, (10) crank shaft speed sensor, (11–14) additional fuel supply system (optional), and (15) boost control system.

In-cylinder pressure measurement was performed with the sensor GU22C made by AVL, designed for thermodynamic analysis. It was a 6.2 mm plug-type sensor that fulfills all reference class requirements when used with the PH04 flame arrestor. Its sensitivity was 36.280 pC/bar, piezoelectric amplifier with offset −8 V, filter 100 kHz, and top dead center (TDC) value 55.000. It included thermally optimized piezoelectric elements that were not influenced by their mounting bores, and therefore the pressure signal was not affected by the mechanical contact between the bore and sensor housing [28]. This was very important, since it has been demonstrated that the housing itself has some impact on the characteristics of ceramic pressure sensors [29].

In-cylinder pressure measurements were related to the crank angle, where two rotations corresponding with the four-stroke working cycle were denoted as the angle between −360° and +360°. Thus, the pressure was registered in the ranges from −360° to −31° and from +90° to +359° with a step of 1°, while in the range from −30° to +89.9° with a step of 0.1°. The in-cylinder pressure values for each angle, obtained in repeated tests, were the subject of further statistical analysis. According to the guide for the expression of uncertainty in measurement [30], the following parameters were considered: standard deviation, range, arithmetic mean or average, and Student’s *t*-distribution quantile for a small number of repetitions.

The test campaign was defined as specified in Table 2.

## 3. Results and Discussion

**Figure 2:**
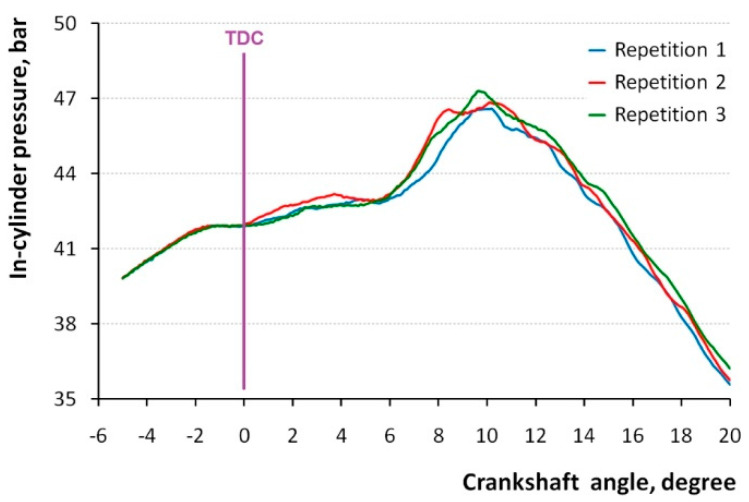
In-cylinder pressure registered in three repetitions in repeatability conditions.

Tests of the measurement systems should be performed in repeatability conditions, i.e., with the same method on identical measurement items, in the same facility, by the same operator using the same equipment within short intervals of time [31]. The described measurement system is highly dependent on all components involved in the fuel mixture preparation, compression, ignition, combustion, decompression, and other stages of the investigated process. Figure 2 presents the example of results of instantaneous in-cylinder pressure measurements from three testing processes, repeated shortly one after another, where all the conditions were kept unchanged as much as possible. Each time it was the last engine cycle with ignition, when the rotational speed was 2499.5 rpm, load torque was zero to keep a steady speed, and injection pressure was 65.3 MPa. The respective temperatures were as follows: air in the collector 17.5 °C, fuel 20.0 °C, engine oil 91.0 °C, and coolant 82 °C. The fuel was injected in two portions, one at 17° before top dead center (BTDC), and the other at 6° BTDC. After this cycle, no fuel entered the cylinder, and therefore the rotational speed gradually slowed down.

Although the pressure diagrams of all three repetitions are essentially the same, substantial differences are seen during the expansion process, especially between angles 5° and 13°. These cannot be attributed to the measurement system, because they are obviously generated by the testing process. Diesel combustion is, in principle, an unsteady turbulent diffusion combustion process [32], and therefore there are cycle variations of the luminosity field and, subsequently, of dynamic in-cylinder pressure and rate of heat release [33]. The turbulent mixing and chemical kinetics of the turbulent combustion process in diesel engines can be coupled with a probability density function [34], which is independent on the measurement system variations. Moreover, in the case of high load, it is also difficult to keep repeatability conditions and to perform a dozen complete test processes in a short time.

Attention should be paid to the fact that before top dead center (TDC), i.e., at angles from −5° to 0°, differences between the repetitions were much smaller than after TDC, where combustion and expansion processes took place. Obviously, statistical analysis of the in-cylinder pressure during the combustion process are heavily influenced by variations of the process itself and provide limited knowledge on the measurement accuracy. In other words, it could be useful for the process analysis, but not for the measurement system assessment. On the basis of the abovementioned results, it was necessary to find a different methodological approach to divide between the process variation and equipment variation. Thus, two measurement conditions were distinguished. In one series, during the test, no more fuel was allowed into the cylinder, but the in-cylinder pressure was still registered in subsequent cycles, while the rotations were slowing down. In the other series, no fuel was involved at all, and the rotations were generated by the starter, while the in-cylinder pressure was registered in a subsequent 32 cycles. Both experiments were repeated three times.

### 3.1. Measurement with No Ignition

**Figure 3:**
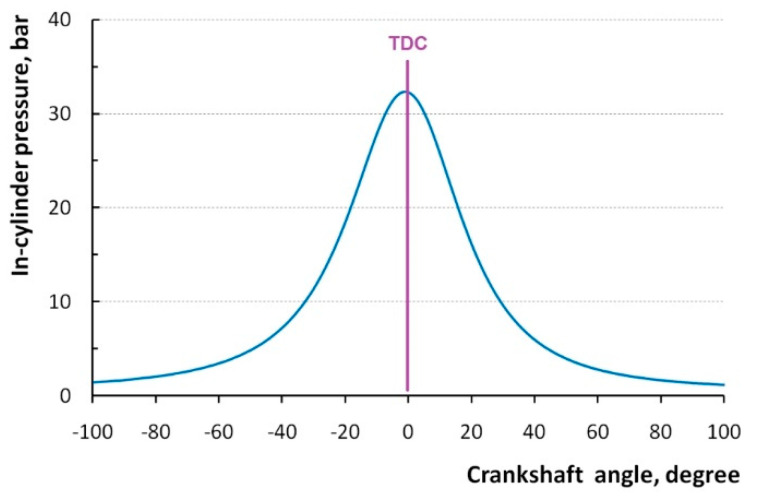
In-cylinder pressure registered in the cylinder without ignition.

A test with no ignition was performed using the starter, which drove the crankshaft. During the cycle, both intake and exhaust valves were closed in the range between −134° and +128°. The diagram in Figure 3 presents the example of pressure measured at each angle from −100° to +100° with respective steps described above. Maximal pressure occurred close to the position of −1°.

**Figure 4:**
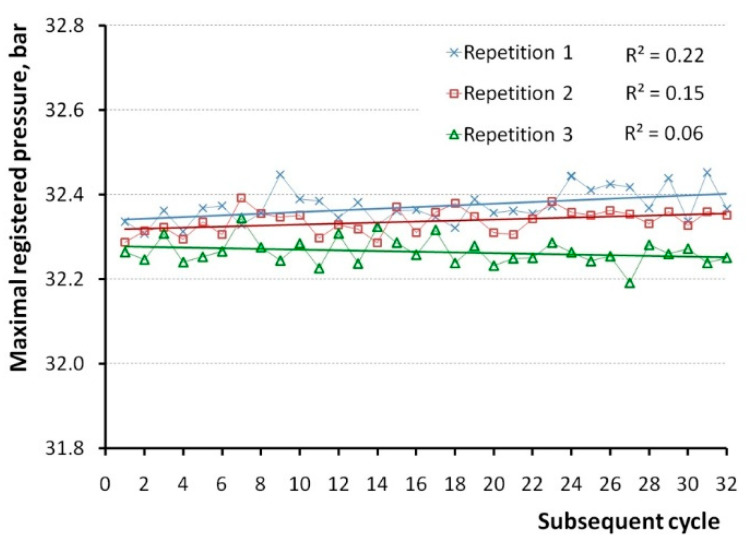
Maximal values of pressure registered in the cylinder without ignition.

Special attention was paid to the maximal in-cylinder pressure in each cycle. During 32 cycles, repeated three times, various maxima were obtained, as shown in Figure 4. The lowest maximal in-cylinder pressure was 32.19 bar, while the highest one was 32.45 bar. The largest difference in maximal pressure between repetitions took place in the cycle No. 27, and it was 0.23 bar.

It should be noted that subsequent repetitions provided generally smaller results of maximal pressure, as shown in Figure 4. Interestingly, the first two repetitions revealed a slight but clear increasing trend, which was not the case for the third repetition. The respective mean values of the obtained maxima from three repetitions are 32.37, 32.34, and 32.26 bar. This demonstrates the inability to keep unchanged the influence of all factors on the measurement results in the time span between repetitions.

**Figure 5:**
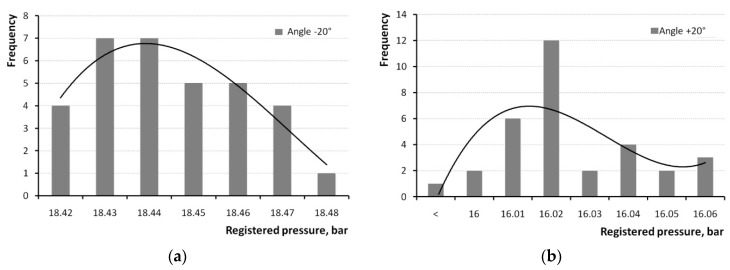
Histograms of in-cylinder pressure measured for angles. (**a**) −20° and (**b**) +20°.

In order to assess repeatability, it is necessary, first, to identify the type of distribution for the pressure variables. For that purpose, histograms were made for angles −20° and +20°, as shown in Figure 5. The distribution type can be assumed to be close to Gaussian.

**Figure 6:**
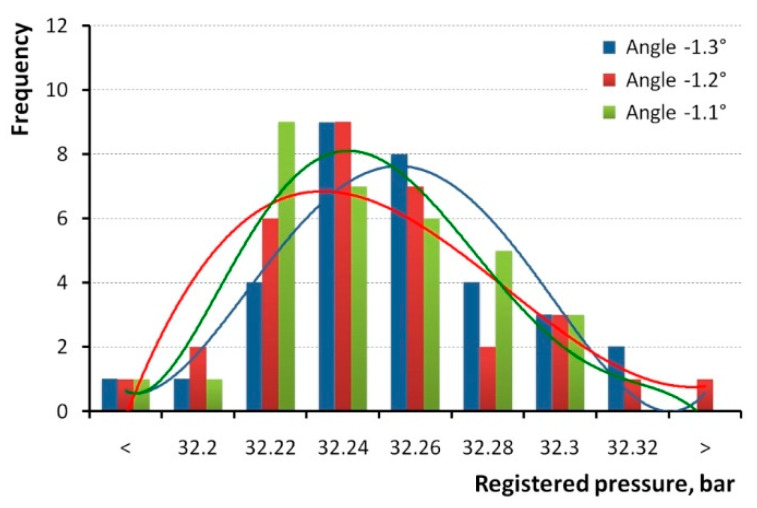
Histograms of in-cylinder pressure measured for angles where maximal pressure appeared.

Interestingly, the maximal in-cylinder pressure of the particular cycle did not always appear at exactly the same angle. In the span of 32 cycles, the maximal pressure was registered at the angles from −0.9° to −1.2° in the first repetition, at the angles from −1.0° to −1.4° in the second repetition, and at the angles from −1.1° to −1.3° in the third repetition. Figure 6 presents respective histograms for the third repetitions, which demonstrate that the distribution can be treated as a normal distribution, despite some asymmetry. The trend lines have a coefficient of determination *R*^2^ = 0.84. 

Hence, to assess uncertainty of the measurement in the examined system, type A methodology can be applied, namely, evaluation of uncertainty by the statistical analysis of a series of observations [30]. Table 3 contains average values and standard deviations for three repetitions during the starter-driven test, 32 cycles each, and expanded uncertainty *U*_0.99_ calculated with coverage factor *k* = 3 for a 99% level of confidence.

Statistically, the average of the three averages can be calculated as $\overset{=}{p_{max}}$ = 3.232 MPa, then, its standard uncertainty can be reduced as follows:

(1) $$u\left(\bar{x}\right)=\frac{u\left(x\right)}{\sqrt{n}},$$

where *n* is the number of repetitions, and in our experiments *n* = 3. Thus, the standard uncertainty $u\left(\overset{=}{p_{max}}\right)$ = 0.02273 MPa, and expanded uncertainty *U*_0.99_ = 0.007 MPa.

### 3.2. Measurement after Ignition

**Figure 7:**
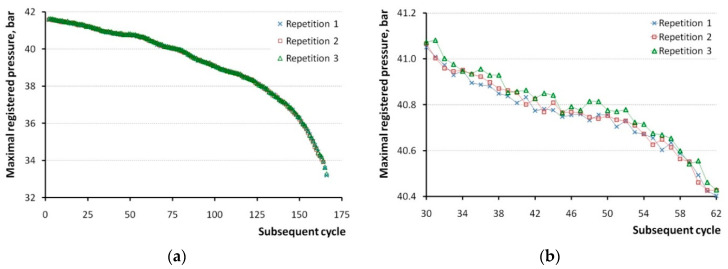
Values of maximal pressure registered in the cylinder after ignition was turned off. (**a**) Entire slow-down process; (**b**) Cycles between 30 and 60.

It was concluded and is shown in Figure 2 above that in-cylinder pressure measurement repetitions with ignition do not fulfill requirements of the repeatability conditions. However, analysis of the subsequent cycles after the last ignition provided interesting results concerning uncertainty of the measurement system. The diagram in Figure 7 presents maximal pressures registered during a subsequent 165 cycles immediately after the last cycle with ignition (post-ignition test).

Unlike the experiments with starter, the pressure in the cylinders in the post-ignition test was continually dropping, very similar to in the three repetitions. Since the maximal difference between the largest and smallest registered values was 0.14 bar, which took place in cycle No. 162, the three graphs in Figure 7a are undistinguishable among one another. Just in the vertical scale of 0.5 bar presented in Figure 7b, it can be seen that, generally, values of the third repetition are slightly larger than that of the first repetition.

Nevertheless, based on the aforementioned experiments with starter, it can be assumed that the distribution is Gaussian, and therefore type A uncertainty can be calculated for the identical cycle number from three repetitions. In that case, Student’s *t*-distribution can be applied, namely quantile *t_α,n_* = 6.956 for *n* = 3 and (1 − *α*) = 0.99. Having uncertainty calculated for $\overset{=}{p_{max}}$ = 3.232 MPa, it was decided to estimate it also for cycles, where registered in-cylinder pressure was ca. 4.15, 4.00, and 3.70 MPa, namely, for cycles No. 1, 70, and 141, respectively. The obtained values are shown in Table 4.

It should be noted that standard uncertainties in this experiment were close to that of the measurements with the starter (Table 3), but because of the small number of repetitions and a large coverage factor *k* = *t_α,n_* = 6.956, the expanded uncertainty *U*_0.99_ was even two times wider.

It should be noted that the abovementioned results are obtained for the repeatable conditions without heavy loads and thermal shocks. We plan to address high-load operation in future studies, but it is noteworthy that the specification of the sensor GU22C provides a value of thermo shock error below 0.2 bar at 9 bar and indicated the mean effective pressure (IMEP) for gasoline. For diesel, it provides a cyclic temperature drift below 0.4 bar at 7 bar IMEP and 1300 rpm. Having determined uncertainty for the cycles without ignition, specification data can be used to estimate combined uncertainty for the measurement under load. However, in order to not overestimate the uncertainty, the issue requires an additional in-depth study in the future. 

### 3.3. Repeatability Tests

It is a common practice to perform repeatability tests for the measurement systems [35,36,37]. Repeatability is the variation produced by the gauge in the process of measuring and is referred to as equipment variation, denoted *EV*. Repeatability is defined as the variation in the results from the same product repeatedly measured in the same laboratory, or, from other perspective, when the same appraiser is repeatedly measuring the same sample in the same environment and obtaining the measurement variation [38]. According to the industrial procedures described in [39], we decided to calculate *EV* values for two combinations:

- Starter test 10 cycle measurements, 3 repetitions;
- Post-ignition test, 10 cycle measurements, 3 repetitions.

Thus, the following formula was applied [39]:

(2) $$\sum E=\sum_{i=1}^{n}\sum_{j=1}^{k}{\left(X_{ij}-X_{i*}\right)}^{2},$$

where it was assumed for the starter test *EV_s_* that *X_i•_* is the average *p*_max_ (unit MPa) obtained from 10 subsequent cycles; *I* is the number of the subsequent cycles from 1 to *n*, in this case *n* = 10; and *j* is the number of repetitions from 1 to *k*, in this case *k* = 3.

Obtained Σ*E* was entered into the equation:

(3) $$s_{E}^{2}=\frac{1}{n\left(k-1\right)}\Sigma E,$$

and, finally, *EV_s_* for a level of confidence of 99% was calculated as follows:

(4) $$EV=5.15s_{E}.$$

Calculated this way, repeatability for the starter test was *EV_s_* = 0.007 MPa, which corresponded with expanded uncertainty *U*_0.99_ estimated for the average pressure. 

Similarly, for the post-ignition test *EV_p-i_*, it was assumed that *X_i•_* is the average *p*_max_ (unit MPa) from 3 measurements in the cycle of the same number; *I* is the number of repetitions for each respective cycle number, *n* = 3; and *j* is the number of the cycles, so that, from 1 to *k*, in that case *k* = 10.

In that case, however, three measurement results were found insufficient for standard deviation calculations, and therefore the range method was applied. It is based on the equation as follows:

(5) $$EV=K_{1}\bar{R},$$

where $\bar{R}$ is the average from 10 obtained ranges, and *K*_1_ = 3.04 is the factor provided in the respective table [39], for a level of confidence of 99%.

In the calculations, the first cycle after ignition was taken, and then the 11th, 21st, etc., up to the 91st cycle. The average range from three repetitions was $\bar{R}$ = 0.0058 MPa, and therefore repeatability of the system from Equation (5) was obtained *EV_p-i_* = 0.018 MPa.

Compared with the starter test repeatability, the post-ignition test provided a ca. 2.5 times larger result. This can be attributed both to the conditions of the measurement and to the substantially smaller number of repetitions. It is important to keep in mind that the post-ignition test operated with the pressure values 3.9–4.2 MPa, which was much closer to the working pressure of 4.7 MPa reached when ignition took place (Figure 2). When the latter is taken as a reference value *RF*, the percent equipment variation *%EV* can be calculated as follows:

(6) $$\%EV=\frac{EV}{RF}\cdot 100\%=\frac{0.018}{4.7}\cdot 100\%=0.4\%.$$

The result of *%EV* = 0.4% obtained from Equation (6) is very positive, since usually the new measurement system is considered to be appropriate for the specific task if its *%EV* lays below 10%.

## 4. Conclusions

While repeatability of the measurement during the combustion process was difficult to estimate even with statistical methods, the proposed original methodology was able to distinguish between variation of the measurement system and that of the unsteady turbulent diffusion combustion process. From the presented tests, the following conclusion can be derived: In-cylinder pressure measurement in an internal combustion engine test bed requires a special approach for the accuracy analysis, since there is no reference device or calibrating masters for the entire system. It was also demonstrated that the variation of results obtained during the ignition process was heavily influenced by the variations of the process itself. Nevertheless, the in-cylinder pressure in the subsequent cycles after the ignition was turned off, met those requirements and could be measured several times in a short time span. Very high repeatability was observed for the cycles driven by the starter, but the maximal pressure values were much lower than during usual tests. 

Using the methodology for type A uncertainty estimation and equipment variation analysis, the accuracy of the system was assessed for both aforementioned cases. The results were *U*_0.99_ = 0.007 MPa for starter-driven mode, and *U*_0.99_ = 0.024 MPa for post-ignition mode. Respective repeatability values were *EV_s_* = 0.007 [MPa] and *EV_p-i_* = 0.018 MPa. The latter value expressed as a percentage of measured pressure was *%EV* = 0.4%, which proved high capability of the evaluated measurement system.

The findings are significant, because they made it possible to distinguish between equipment variation and any additional kind of in-cylinder high-pressure results’ dispersion. In future studies, we plan to focus on repeatability in high-load operating conditions, and especially under thermal shocks.

## Figures and Tables

**Table 1:** Basic technical characteristics of the AVL 5402 engine.

Number of Cylinders	1
Bore	85.01 mm
Stroke	90.00 mm
Displacement	511.00 cm^3^
Combustion type	Compression ignition
Valve system	4 valves
Compression ratio	17.0 ÷ 17.5
Fueling system	Direct injection, single injector, Common rail system
Maximum effective power, without supercharging	6 kW
Maximum effective power, with supercharging	16 kW
Rated engine speed	4200 min^−1^
Injection pressure	180 MPa

**Table 2:** Test campaign definition.

Item	Investigated Configuration	Objectives	Parameter	Possible Constraints
In-cylinder instantaneous pressure measurement with sensor GU22C	Cycle with ignition Immediate cycles after ignition Starter-driven cycles	Assessment of the measurement accuracy	Standard uncertainty, expanded uncertainty, repeatability *EV*	Turbulent combustion process, environmental conditions

**Table 3:** Obtained uncertainties for three repetitions with a starter.

	Number of Cycles	Average $\bar{p_{max}}\;(\mathrm{MPa})$	Range *R* (MPa)	Standard Uncertainty *u* (MPa)	Expanded Uncertainty *U*_0.99_ (MPa)
Repetition 1	32	3.237	0.014	0.003936	0.012
Repetition 2	32	3.234	0.011	0.002855	0.009
Repetition 3	32	3.226	0.015	0.003157	0.009

**Table 4:** Uncertainty estimation for three different cycles.

Subsequent Cycle No.	Average $\bar{p_{max}}\;(\mathrm{MPa})$	Range *R* (MPa)	Standard Uncertainty *u* (MPa)	Expanded Uncertainty *U*_0.99_ (MPa)
1	4.159	0.006	0.003086	0.021
70	4.011	0.003	0.001322	0.009
141	3.704	0.007	0.003420	0.024
